# Interceptive Orthodontics and Growth Modification Therapy with Fixed Functional Appliance: A Case Report

**DOI:** 10.5005/jp-journals-10005-1120

**Published:** 2011-04-15

**Authors:** MS Ravi, Jatin Ahuja

**Affiliations:** 1 Professor, Department of Orthodontics, AB Shetty Dental College, Karnataka, India; 2 Orthodontic Practice, Shivaji Park, Punjabi Bagh, New Delhi, India

**Keywords:** Space regaining, Growth modification, Fixed functional appliance.

## Abstract

A 12-year-old female patient presented with proclined upper anteriors on a class II skeletal base, a retrognathic mandible and high maxillary- mandibular plane angle. Lower first molars were mesially tipped and lower second premolars were impacted. Treatment plan included uprighting and distalising the lower molars followed by growth modulation with Jasper Jumper to correct the mandibular retrognathism. Final finishing and detailing of occlusion was carried out through 0.022” MBT prescription preadjusted edgewise appliance therapy.

## INTRODUCTION

A 12-year-old female patient presented with a chief complaint of proclined upper anterior teeth. There was no relevant family history. No history of any habits and the patient was positively inclined for treatment. The extraoral examination suggested a convex profile, deficient mandible, normal maxilla in sagittal plane, incompetent lips and mild increase in lower facial height. Intraorally, the patient was in mixed dentition period having deciduous canines and second molars in the upper arch. The lower arch had all permanent teeth except second premolars and third molars. The upper arch was constricted and asymmetric. Lower arch too was constricted; the 1st molars were mesially tipped with loss of second premolar space on both the sides.

**Figs 1A to E:**
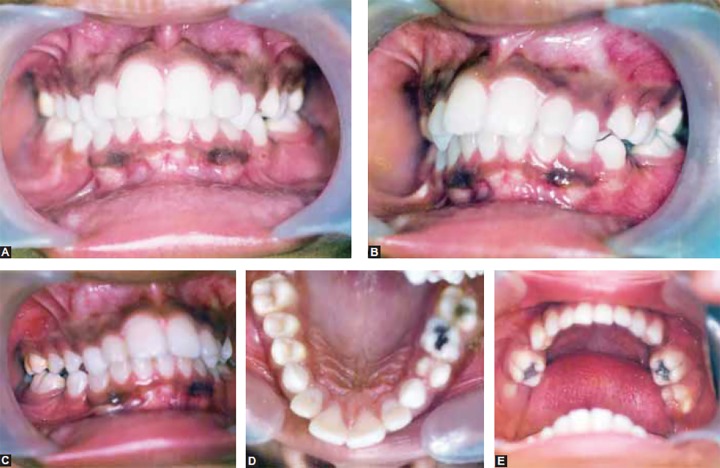
Pretreatment intraoral photographs

**Figs 2A to C:**
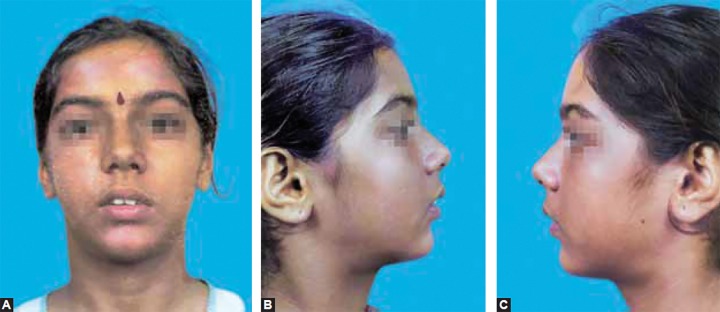
Pretreatment extraoral photographs

Upper and lower molars were in class I relationship, the canines were in class II relationship. Overjet was 10 mm and overbite was 6 mm. The midlines were coincident (Figs 1A to 2C).

**Figs 3A and B:**
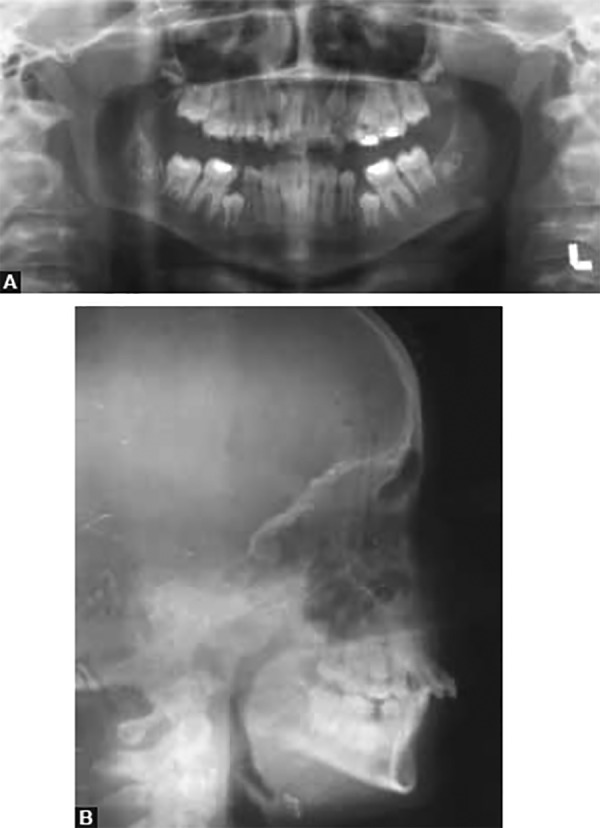
Pretreatment radiographs

Orthopantomogram revealed presence of all unerupted teeth, mesially tipped lower first molars blocking the eruption of second premolars and favorably placed upper canines and left second premolar (Fig. 3A).

Cephalometric analysis confirmed the clinical findings (Fig. 3B). Patient had a skeletal class II pattern with retrog-nathic mandible. Maxillary-mandible plane angle was near normal with mild increase in lower facial height. Upper incisors were proclined, the lower incisors were upright and the interincisal angle was reduced. Jaraback ratio indicated a favorable growth pattern. The lip position were also normal in relation to the Rickett’s esthetic plane (Table 1).

## TREATMENT PLAN

- Oral prophylaxis
- Uprighting the lower 1st molars and space regaining for the eruption of 2nd premolars with modified lingual arch and NiTi coil springs
- Mandibular advancement with Jasper Jumper, a fixed functional appliance
- Fixed appliance therapy for occlusal detailing and finishing with 0.022” MBT preadjusted edgewise appliance
- Long-term retention plan with upper and lower Hawley’s appliances.

The lower first molars are required to be uprighted to allow the eruption of second premolars. This would also harmony with normal skeletal and dental relationships result in establishing the class II molar relationship in a class II skeletal relationship. The establishment of molar class II relationship would be a favorable situation during the skeletal advancement procedure. Mandibular advancement with a fixed functional appliance is ideal as the patient ^1,2^ is in her near completion stage of growth spurt. The prognosis of the case would be good with the establishment of structural stability and harmony.

## TREATMENT PROGRESS

**Figs 4A to D:**
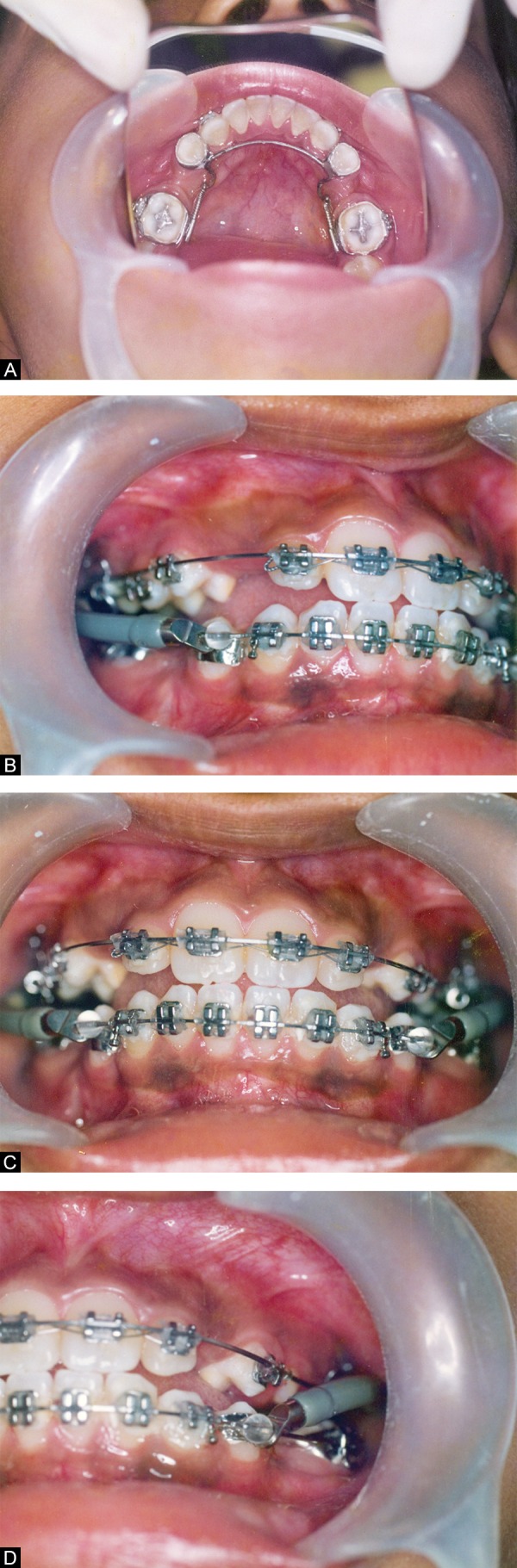
Appliances in place

The lower lingual arch fabricated in the 0.036” SS wire, consisted of a modified U loop mesial to lingual sheath on the molar (Fig. 4A). The arch was soldered on to the 1st premolar bands for additional anchorage. 0.016” NiTi push coil springs were incorporated into the lingual arch so as to provide the uprighting and space regaining force onto the 1st molars. 0.022” MBT PEA was bonded and initial aligning and leveling was carried out using 0.016” NiTi arch wires. The arch wires were sequentially progressed into heavier dimension till a full size wire of 0.021 × 0.025” SS wire could be engaged. The NiTi coil springs were activated once in every 6 weeks and the uprighting of molars progressed favorably.

The process of uprighting, space regaining and initial aligning of all teeth were completed in 8 months of treatment. Jasper Jumper of size 30 was selected and fixed on either side (Figs 4B to D). Reactivation of Jasper Jumper was required after a period of 8 weeks and molar class I with skeletal class I relationship was established in about 22 months of total treatment time.

**Figs 5A to E:**
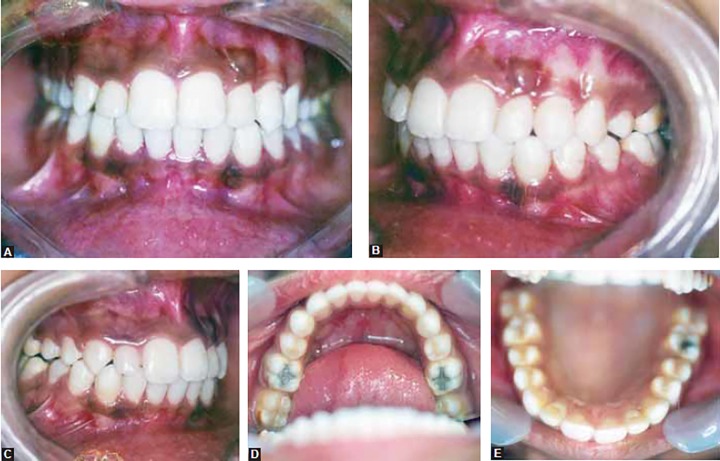
Posttreatment intraoral photographs

The upper canines and premolars and the lower 2nd premolars were erupting into the arch. These teeth were bonded and included in the arch wire and were aligned using a 0.016” NiTi piggy back wire. The final occlusal finishing and detailing were carried out with TMA arch wires and elastics (Figs 5A to E). Long-term retention with upper and lower Hawley’s appliance was advised.

**Figs 6A to C:**
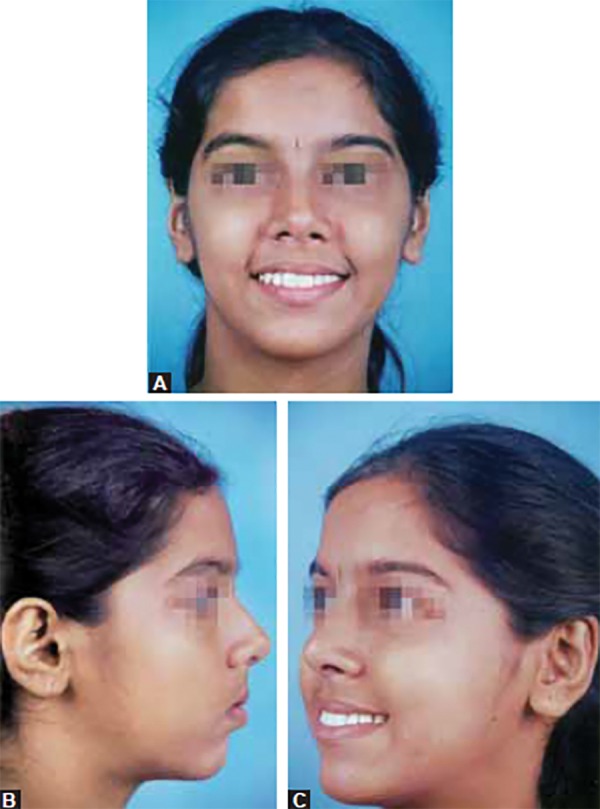
Posttreatment extraoral photographs

At the end of 28 months of active treatment, all the obj ec-tives of the treatment could be achieved (Figs 6A to C). Good functional occlusion, esthetics and structural harmony and stability were established.

## CONCLUSION

**Figs 7A and B:**
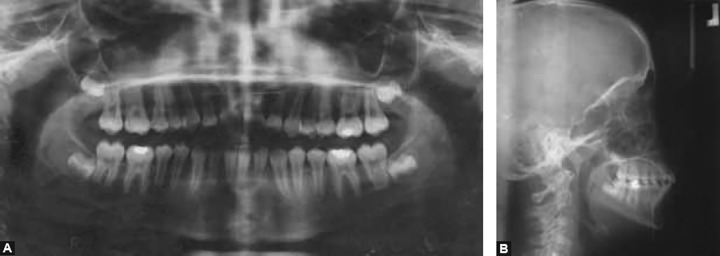
Posttreatment radiographs

The growth modification with the fixed functional appliance of Jasper Jumper for a female patient who was in postpuber-tal growth period^3^ has been successful in establishing the skeletal class I relationship by the mandibular advancement (Table 1). The lower incisors were still upright to the basal bone with no appreciable changes in the ratio of anterior to posterior facial height. The buccal segment occlusion was significantly improved with molar and premolars in good functional occlusion. The canines were in class I relationship, the overjet is reduced to 3 mm and the overbite was normal. All unerupted teeth, including the impacted lower 2nd premolars, were allowed to erupt and aligned into the occlusion (Figs 7A and B).

**Table 1:** **Table1:** Cephalometric analysis

*Variable*		*Pre-* *Variable*		*Post-* *Variable*		*Changes*	
SNA		83°		83°		0	
SNB		75°		78°		+3°	
ANB		8°		5°		–3°	
Wits appraisal		9 mm		7 mm		2 mm	
U1 -Mx plane		115°		103°		–12°	
L1 -Mn plane		92°		95°		+3°	
Interincisal angle		120°		128°		+8°	
Mx-Mn plane		32°		34°		+2°	
Upper ant. facial ht.		46 mm		46 mm		0	
Lower ant. facial ht.		63 mm		69 mm		6°	
Face ht. ratio		58%		60%		2%	
L1-A-Pog.line		+1 mm		+3 mm		+2 mm	
Lower lip -E plane		0 mm		+2 mm		+2 mm	
Angle N-S-Ar		125°		122°		–3°	
Angle S-Ar-Go		137°		140°		–3°	
Angle Ar-Go-Gn		135°		134°		–1°	
Total of 3 angles		397°		396°		–1°	
U-Go angle		60°		57°		–3°	
L-Go angle		75°		77°		+2°	
Y-Axis		70°		70°		0	
S-Go		67 mm		70 mm		+3 mm	
N-Me		109 mm		116 mm		+3 mm	
Ratio		61.4 %		60.3%		–1%	

**Table 2:** **Table 2:** Treatment outcome assessment

*Parameter*				*Pretreatment*		*Posttreatment*		*Change*	
Index of treatment need (IOTN)		Dental health component		51		1		–	
		Esthetic component		6		1		–	
Peer assessment rating (PAR)				31		3		28 (90.3%)	

**Figs 8A to C:**
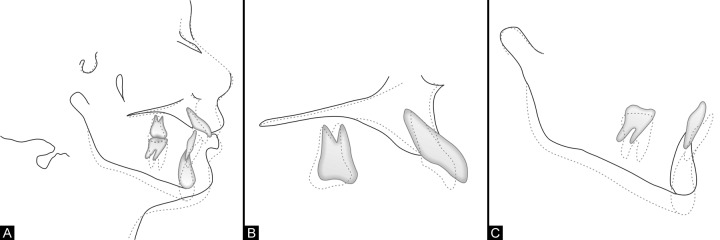
Cephalometric superimpositions

The evaluation of changes brought about by the growth modification procedure along with fixed appliance therapy (Tables 1 and 2) indicates a significant improvement in the overall dental occlusion, facial esthetics along with structural balance and harmony (Figs 8A to C).
